# Age Interactions in the Development of Naturally Acquired Immunity to *Plasmodium* *falciparum* and Its Clinical Presentation

**DOI:** 10.1371/journal.pmed.0040242

**Published:** 2007-07-31

**Authors:** John J Aponte, Clara Menendez, David Schellenberg, Elizeus Kahigwa, Hassan Mshinda, Penelope Vountasou, Marcel Tanner, Pedro L Alonso

**Affiliations:** 1 Barcelona Centre for International Health Research, Hospital Clinic/IDIBAPS, Universitat de Barcelona, Barcelona, Spain; 2 Centro de Investigacão en Saude de Manhica, Manhica, Mozambique; 3 Ifakara Health Research and Development Centre, Ifakara, Tanzania; 4 Swiss Tropical Institute, Basel, Switzerland; Rigshospitalet, Denmark

## Abstract

**Background:**

Naturally acquired malaria immunity has many determinants and, in the absence of immunological markers of protection, studies assessing malaria incidence through clinical endpoints remain an approach to defining immunity acquisition. We investigated the role of age in disease incidence and the effects of chemoprophylaxis on clinical immunity development to Plasmodium falciparum during a randomised controlled trial.

**Methods and Findings:**

A total of 415 Tanzanian infants were randomly assigned to receive weekly malaria prophylaxis with Deltaprim (3.125 mg of pyrimethamine plus 25 mg of dapsone) or placebo between the ages of 2 and 12 mo. Children were followed up until 4 y of age. Uncomplicated febrile malaria, severe malaria, and anaemia morbidity were assessed through hospital-based passive surveillance. Compared with the group of control participants, there was a marked reduction in the incidence of clinical malaria, severe malaria, and anaemia in the group of children who had received chemoprophylaxis during the first year of life. After discontinuing the intervention, there was a significant increase in the incidence of clinical malaria for 2 y. The cumulative rates of clinical malaria, by age 4 y, were slightly higher in the group of children who had previously received chemoprophylaxis: 3.22 episodes versus 3.02 episodes in the group of control participants; rate difference 0.20 (95% confidence interval [CI]: −0.21 to 0.59). By age 4 y, the cumulative rates of severe malaria, however, were slightly lower in chemosuppressed children (0.47 versus 0.59) (rate difference −0.12 [95% CI: −0.27 to 0.03]). The number of episodes of anaemia was also slightly lower in chemosuppressed children by age 4y: 0.93 episodes (95% CI: 0.79 to 0.97) versus 1.12 episodes in the group of control participants (95% CI: 0.97 to 1.28) (rate difference −0.19 [95% CI: −0.40 to 0.01]), respectively.

**Conclusions:**

Reducing exposure to P. falciparum antigens through chemoprophylaxis early in life can delay immunity acquisition. Infants appear to acquire immunity faster than older children, but have a higher risk of developing severe forms of malaria and anaemia. These findings provide insight on the interplay between immunity and exposure-reduction interventions.

## Abstract 2

John Aponte and colleagues report that reducing exposure to parasite antigens early in life through chemoprophylaxis, while reducing the incidence of severe malaria, can delay the development of immunity.

## Editors' Summary

**Background.:**

Malaria is a life-threatening disease caused by *Plasmodium falciparum,* a parasite that is transmitted from person to person by infected mosquitoes. People living in areas where malaria is common (malaria-endemic regions) gradually acquire immunity to this parasite through repeated exposure to it, but the development of this immunity is complex. As with other infectious diseases, the immune system has to learn how to recognize and attack the parasite so that parasitaemia (the number of parasites in the blood) is kept to a minimum. In addition, because several symptoms of clinical malaria (for example, the characteristic fever) are side effects of the immune system's response to the parasite, it also has to learn not to overreact to this foreign invader. Consequently, in malaria-endemic areas, where individuals first become infected with P. falciparum as infants, many children develop severe malaria and anemia (parasite-induced killing of red blood cells) during their first 5 y of life. Later, as their immunity to P. falciparum develops, they experience less severe disease and reduced parasitaemia.

**Why Was This Study Done?:**

Little is known about how quickly immunity to P. falciparum is acquired or how age affects this process. This study was done to document the long-term effects of malaria prophylaxis given to young Tanzanian infants. More specifically, the researchers examined whether and how prophylaxis affects the rate of acquisition of malaria immunity, and whether a possible delay in immunity would be associated with more severe forms of malaria in children.

**What Did the Researchers Do and Find?:**

Infants in Ifakara, a town in Tanzania, received either antimalarial tablets or placebo (inactive) tablets every week between the ages of 2 and 12 mo. Cases of clinical malaria (parasitaemia and fever), severe malaria (parasitaemia plus dehydration, breathing problems, impaired consciousness, or low blood sugar), and anemia among the children were recorded until they were 4 y old. During their first year, the children receiving antimalarial drugs had fewer episodes of malaria, severe malaria, and anemia than the children receiving the placebo. After the antimalarial drugs were stopped, cases of clinical malaria, severe malaria, and anemia were more frequent among the treated children than among the control children for the next 2 y. By the time they were 4 y old, the treated children had had slightly more episodes of clinical malaria in total than the control children, but slightly fewer episodes of severe malaria and anemia.

**What Do These Findings Mean?:**

These findings show that reducing exposure to P. falciparum in early life with continuous malaria prophylaxis delays the acquisition of immunity to the parasite. They also show that infants acquire this immunity slightly faster than do older children, but that this speedier acquisition of immunity is accompanied by a slightly increased risk of severe malaria and anemia. Because these findings may, in part, reflect a local decrease in malaria transmission that occurred during the study, they need confirming in other settings and with many more children. Nevertheless, they suggest that although age at first exposure to P. falciparum helps to determine both the rate at which immunity is acquired and the severity of disease caused by the parasite, delaying the acquisition of immunity through early prophylaxis is unlikely to have long-term negative health effects. This information will help public-health experts to plan prophylactic interventions designed to control malaria transmission in endemic regions.

**Additional Information.:**

Please access these Web sites via the online version of this summary at http://dx.doi.org/10.1371/journal.pmed.0040242.

- US Centers for Disease Control and Prevention information on malaria and on immune responses to malaria (in English and Spanish)
- MedlinePlus encyclopedia page on malaria (in English and Spanish)
- Information from the World Health Organization on malaria (in English, Spanish, French, Russian, Arabic, and Chinese)
- Short articles from the Wellcome Trust on immunity to malaria and on malaria and the human immune system

## Introduction

More than 100 y ago on the island of Java, Koch described how the clinical and parasitological manifestations of malaria decreased with age. He interpreted this as a function of acquired resistance through exposure to the parasite [1]. However, despite a plethora of immunological investigations, no surrogate marker of protective immunity has been identified. A number of humoral and cellular immune responses are known to be associated with reduced risk from Plasmodium falciparum infection and disease [2], but none has been shown to be causally related. Hence, studies assessing malaria incidence through its different clinical endpoints remain the best approach to determine the rate of acquiring immunity.

The relationship between the risk of malaria—as a surrogate of acquired immunity—and age is confounded by the intensity of transmission, because people living in high-transmission areas will tend to be exposed at younger ages. Hence, it is unclear as to whether it is the frequency of infection or age itself which is the prime determinant of the clinical manifestations. To our knowledge, few studies have had the opportunity to investigate the effect of age on the development of malaria immunity. Perhaps the best information comes from a descriptive study of parasitaemia in non-immune migrants to malaria-endemic areas of Irian Jaya, which suggested that adults acquired sterilizing immunity more rapidly than children [3].

Information is also available on the age-related changes in the manifestations of clinical malaria, principally cerebral malaria and anaemia [4]. Malaria-associated anaemia is typically described in younger children (i.e., <2 y) [5]. Historically, older children are also suspected to have a higher risk of cerebral malaria, but this age dependency is less well defined, owing partly to the difficulties in objectively assessing the neurological status in infants.

In our opinion, the effects of a limited period of suppression of malaria infection on the development of naturally acquired immunity have been insufficiently addressed by current research. The most complete evaluation of potential risks was carried out in the Gambia in the context of a placebo-controlled intervention trial [6]. Maloprim was administered for 5 y consecutively during the transmission season to children aged between 6 mo and 5 y. The study showed that there was a small, though not statistically significant, increase in the probability of dying during the year after chemoprophylaxis was stopped, but that chemoprophylaxis was associated with an overall improvement of survival [7].

A similar result was observed for malaria attacks. Malaria-specific antibody levels at the end of chemoprophylaxis and 1 y later were lower, but the lymphoproliferative responses to malaria antigens were increased in children who had received chemoprophylaxis (chemosuppressed group) [8,9]. More recently, a study in Tanzania gave infants malaria chemoprophylaxis with Deltaprim. The results of the intention-to-treat analysis showed that malaria chemoprophylaxis had an efficacy of 60.5% and 57.3% in reducing the incidence of clinical malaria and severe anaemia, respectively, up to the age of 13 mo. However, after stopping chemoprophylaxis, the risks of both outcomes increased in the previously chemosuppressed group until the age of 18 mo, by 1.8 and 2.2, respectively [10].

Our study reports the effects of malaria chemoprophylaxis given during the first year of life on malaria, severe malaria, severe anaemia, and general morbidity during 4 y of clinical follow-up. Improved understanding of the development and determinants of anti-malaria immunity and the ability to cope with severe manifestations will not only help the rational design of vaccines, but will provide information as to the most appropriate use of malaria-control tools.

## Methods

### Study Area

The study was conducted between 1995 and 1999 in Ifakara, a town in the Kilombero District of the Morogoro region in Tanzania. The demographic and geographic characteristics of the area have been described in detail elsewhere [10]. At the beginning of the study, P. falciparum malaria transmission in the area was highly endemic; the average entomological inoculation rate (EIR) in a nearby village was about 300 infective bites per person per year [11]. During the course of this study, the malaria epidemiology changed [12], and an EIR of 29 (95% confidence interval [CI]: 19 to 44) was documented in 2000 [13]. St. Francis Designated District Hospital (SFDDH), a 370-bed facility, and its adjacent maternal and child-health clinic serve the town. A number of pharmacies and health dispensaries have also opened since deregulation of the health sector in the late 1990s. Malaria control during the study period relied on the prompt treatment of presumptive episodes with chloroquine. However, the prevalence of parasite resistance to chloroquine was very high (approximately 70% parasitological failure at day 7) [14]. The use of mosquito nets increased in the area from about 51% in 1996 to 71% in 2001. Malaria was responsible for 37% of all paediatric admissions to SFDDH in 1995, with a 3% case-fatality rate. There was a marked age dependence of malaria disease and death; 44% of cases and 54% of the inpatient malaria deaths occurred in children aged less than 1 y [5]. Severe anaemia (packed cell volume [PCV] <25%) was also an important cause of hospital admission and death; 41% of children aged less than 1 y were admitted with severe anaemia and, of these, more than 53% received a blood transfusion [5].

### Study-Design Population

In January 1995, a four-arm randomised placebo-controlled trial began assessing the efficacy of malaria chemoprophylaxis with weekly Deltaprim (3.125 mg of pyrimethamine plus 25 mg of dapsone) and/or iron supplementation between 2 and 12 mo of age for the prevention of malaria and anaemia in infants. Details of the study design and results during the first 18 mo of follow-up have been described elsewhere [10]. Only infants whose mothers had given written informed consent took part in the study. Research and ethical clearance was granted by the Medical Research Coordination Committee of the National Institute for Medical Research through the Tanzanian Commission of Science and Technology and the Hospital Clinic Ethics Committee in Barcelona, Spain. The present analysis is based on the incidence of overall and severe malaria morbidity, as well as severe anaemia, detected through the hospital-based passive case-detection system during the 4-y period following recruitment. In order to avoid any possible confounding effect of the iron supplementation, the two cohorts of children receiving iron are not included in this analysis. The long-term effects and safety of iron supplementation are presented elsewhere (Menendez et al., unpublished data).

### Morbidity Surveillance

Since 1994, SFDDH and its adjacent maternal and child-health clinic have used a round-the-clock, passive case-detection surveillance system. Project officers issued a questionnaire, which recorded a patient's main symptoms and signs upon admission, and they also conducted basic laboratory investigations for all children who were ill. A unique identification card given to all study participants (children who had received chemoprophylaxis [chemosuppressed group] as well as control participants) at recruitment allowed their identification during all contact with the project personnel. A system of bi-monthly demographic surveillance provided information on vital events not registered at the hospital. Malaria smears were prepared for all inpatients and for out-patients when there was fever, history of fever, or pallor.

### Laboratory Methods

Thick blood films were stained with Giemsa and read on a light microscope following standard quality-control procedures [15]. The PCVs were measured using a micro-centrifuge and a Hawksley haematocrit reader (http://www.hawksley.co.uk/).

### Statistical Methods and Definitions

Clinical malaria was defined as an episode of fever (axillary temperature >37.5 °C) plus P. falciparum parasitaemia of any density at any contact with the health services. Severe anaemia was defined as a PCV of less than 25% at any contact with the health services. Severe malaria was defined as the presence of parasitaemia together with any of the following risk factors: dehydration, respiratory distress, impaired consciousness, and hypoglycaemia. These factors were selected because they had been previously shown to be independently associated with death for children in the study setting [5]. In order to avoid double counting of episodes, children who had been treated for clinical malaria, severe malaria, or severe anaemia were considered not to be at risk for a new episode during a 28-d period after beginning treatment. For each outcome (clinical malaria, severe malaria, and severe anaemia), a monthly incidence was calculated using the number of episodes divided by the time during which the patient had been at risk and expressed as episodes per person-years at risk (PYAR) for the two groups.

The effect of chemoprophylaxis on the risk of clinical malaria and anaemia changes with age, and is different before and after the period of chemoprophylaxis. Thus, in order to produce smooth estimates of the effect of chemoprophylaxis with age, we modelled the incidence of clinical malaria, severe anaemia, and other outcomes using Poisson regression models of small time intervals, and imposing Bayesian autoregressive constraints. This type of model allows the effect of chemoprophylaxis to change with time but, for any given time period, the effect should be similar to the effect in the previous period of time. Details of the models are presented in Protocol S1.

In order to compare the occurrence of malaria, anaemia, or other outcomes, we calculated the cumulative rate (CR) as the area under the curve for the estimated monthly incidence in each treatment group. The CR at any given age could be interpreted as the expected number of episodes that a child experiences up to that age. Details of the CR estimation are presented in Protocol S1. The graphs were produced using Stata version 8.0 (Stata Corporation, http://www.stata.com/) software. For the Bayesian models, we present the mean posterior value and the 95% CIs. Bayesian models were fitted using WinBugs 1.4 [16] and the Bugs.R procedure [17].

## Results

**Figure 1:**
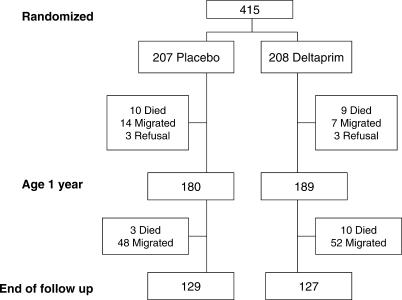
Trial Profile

The trial profile for the intervention and corresponding cohort of control participants is shown in Figure 1. Of the original 415 children, 256 (61.7%) completed the 4 y of follow-up. Thirty-two children (7.7%) died, 121 (29.2%) migrated or were lost to follow-up, and six (1.4%) refused to continue in the study.

### Risk of Clinical Malaria

**Figure 2:**
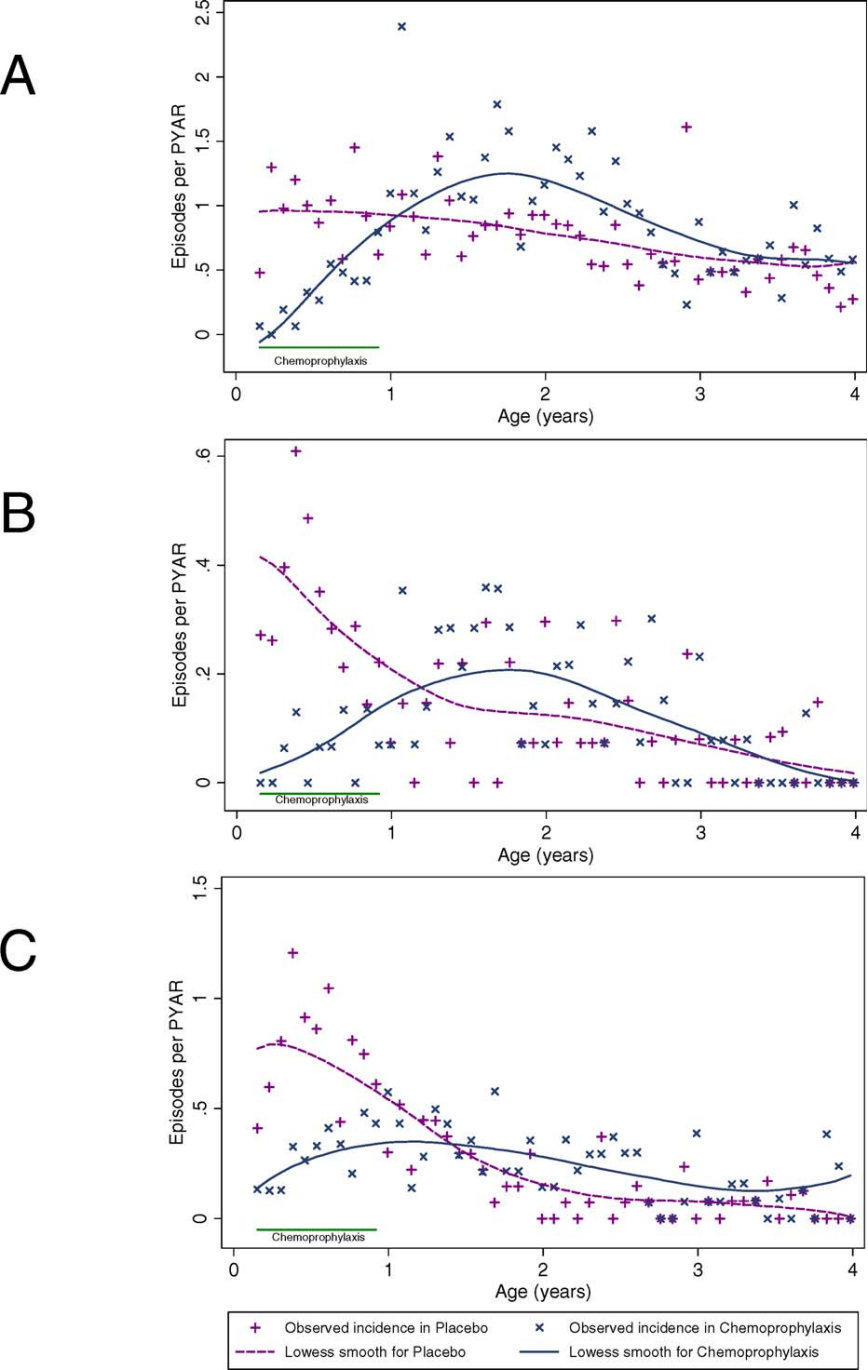
Incidence of Clinical Manifestations of Malaria by Group and Age (A) Clinical malaria, (B) severe malaria, and (C) severe anaemia. Episodes per PYAR are shown.

**Figure 3:**
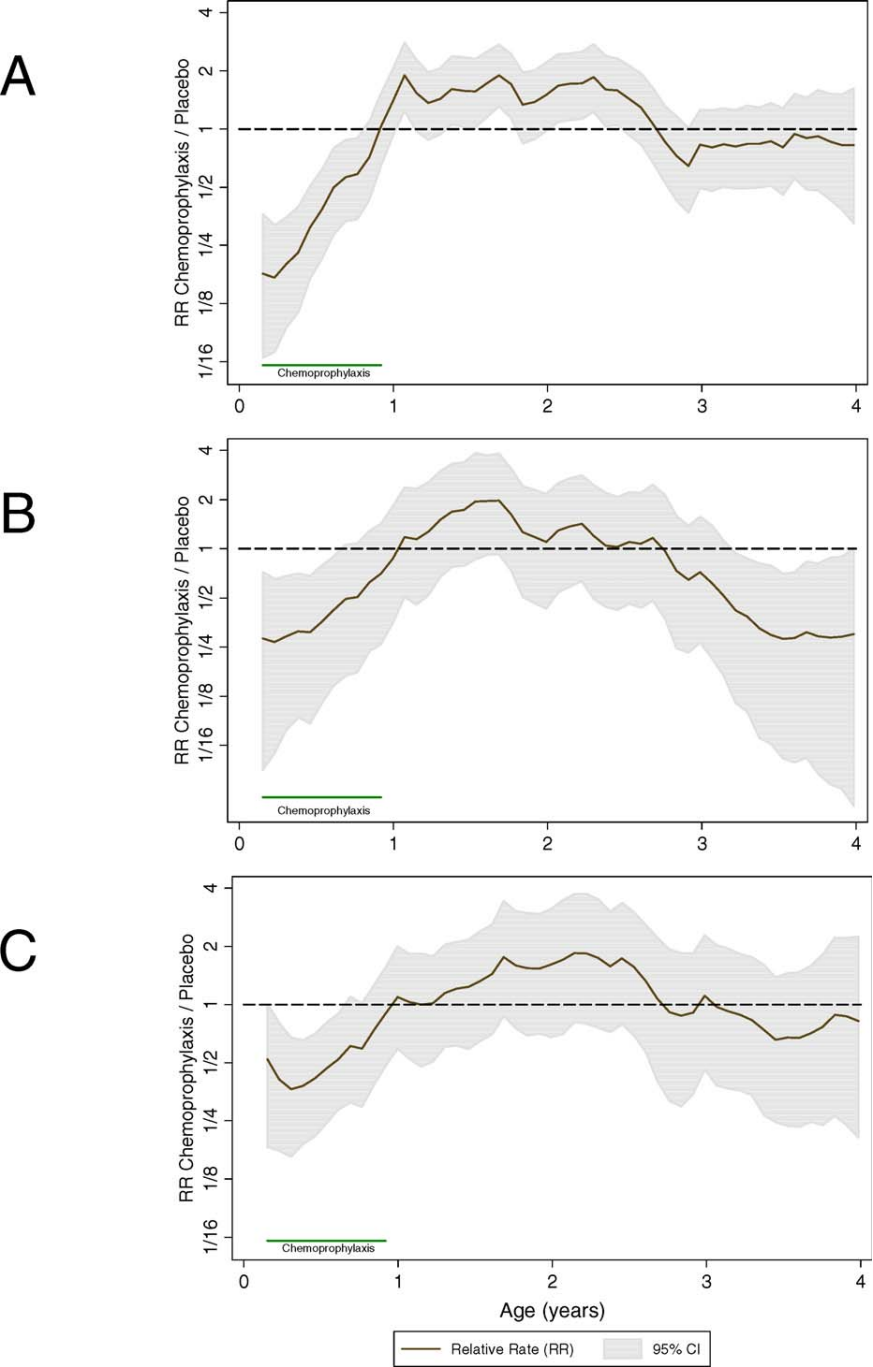
Relative Rates of Clinical Manifestations of Malaria by Age (A) Clinical malaria, (B) severe malaria, and (C) severe anaemia. Relative-rate values higher than 1 indicate a higher incidence in the chemoprophylaxis group, whereas relative-rate values lower than 1 indicate a higher incidence in the placebo group.

**Figure 4:**
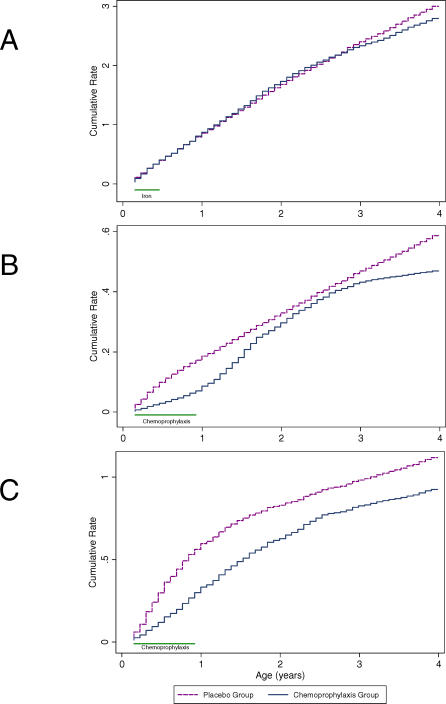
CRs of Clinical Manifestations of Malaria by Group and Age (A) Clinical malaria, (B) severe malaria, and (C) severe anaemia.

Figures 2–4 present the relationship between the different outcomes and age. The age-specific monthly incidence of clinical malaria in the two cohorts is shown in Figure 2A. The malaria risk in children receiving placebo declined slowly during the first 4 y of life, from 0.96 episodes/PYAR in children aged between 2 and 12 mo of age to 0.72 episodes/PYAR in children aged between 1 and 4 y (Table 1). The risk of malaria during the first year of life was dramatically reduced to 0.31 episodes/PYAR by chemoprophylaxis, although there was a clear tendency for the size of this effect to decrease as the infants became older. Once prophylaxis was stopped, chemosuppressed children had a significantly higher rate of clinical malaria compared with their contemporary control participants, and this higher rate lasted for at least 18 mo (Figure 3A).

**Table 1:**
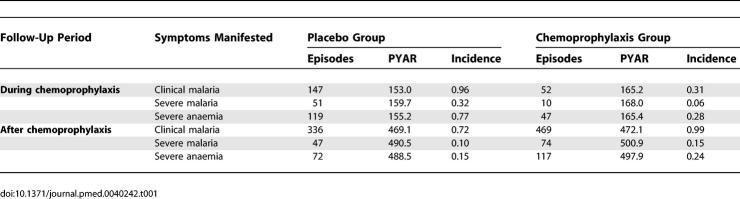
Incidence of Clinical Manifestation of Malaria by Follow-Up Period

Figure 4A shows the CR for clinical malaria in the two cohorts. The advantage gained by the group of children who had received chemoprophylaxis during the first year of life was lost within the following 12 mo. At the end of the follow-up period, the CR was slightly higher in the chemosuppressed group versus the group of control participants; 3.22 episodes versus 3.02 episodes, CR difference of 0.20 (95% CI: −0.21 to 0.59).

### Risk of Severe Malaria

Figure 2B shows the monthly incidence of severe malaria between the two cohorts. Among children who were chemosuppressed during their first year of life, the distribution of severe malaria episodes is shifted to the right; by the age of 4 y, they experience an incidence rate of 0.32 episodes/PYAR. Children in the placebo group (control participants) had an average of 0.59 (95% CI: 0.48 to 0.71) episodes of severe malaria (Table 1) by the age of 4 y. Figure 3B shows the evolution of the relative rate of having a severe malaria episode during the 4 y of follow-up. As with the pattern for clinical malaria, the rebound in the risk of severe malaria starts immediately after discontinuing prophylaxis, but in this case is shorter, lasting for approximately 1 y. Thereafter, there is a tendency for the risk of severe malaria to be lower in the chemosuppressed group, but the difference is not significant. By the end of the follow-up period at the age of 4 y, the CR of severe malaria episodes was slightly lower in the intervention cohort of children who had received chemoprophylaxis during the first year (0.59 versus 0.47), with a rate difference of −0.12 (95% CI: −0.27 to 0.03) (Figure 4B).

### Risk of Severe Anaemia

Figure 2C shows the monthly incidence of severe anaemia. On average, by the age of 4 y, children will have had 1.12 (95% CI: 0.97 to 1.28) episodes of anaemia with a PCV of <25%; nearly half these episodes are in children aged less than 1 y (Table 1). This corresponds to an incidence rate of 0.77 episodes/PYAR.

Figure 3C compares the relative rate of developing anaemia up to the age of 4 y in the two cohorts. During prophylaxis, the anaemia rate was significantly lower in the chemosuppressed group, but there was a tendency for the efficacy of the intervention to decrease over time. Thereafter, there was an increased risk of anaemia in the intervention group that did not equalize until around 30–36 mo of age. Figure 4C shows that the magnitude of the rebound for anaemia is relatively small. By the age of 4 y, chemosuppressed children had experienced a marked reduction in the risk of anaemia, and had a reduced CR (0.93 episodes [95% CI: 0.79 to 0.97] versus 1.12 [95% CI: 0.97 to 1.28]), corresponding to an overall difference of −0.19 (95% CI: −0.40 to 0.01).

### Risk of Hospital Admissions

Children in the placebo group had, on average, 3.3 hospital admissions during the first 4 y of life (95% CI: 3.03 to 3.60), compared to 3.1 admissions (95% CI: 2.83 to 3.37) for children who had received chemoprophylaxis during the first year. The admission rate was highest in infants and decreased sharply with age (incidence rate of 1.25 admissions/PYAR).

## Discussion

This study investigated the development of clinical immunity against malaria during the first 4 y of life in two contemporaneous cohorts of children experiencing P. falciparum infections at different ages. A major strength of this study was that, although transmission intensity varied over the course of follow-up, both cohorts were similarly exposed to malaria. The incidence of clinical malaria in children receiving placebo fell gradually during the first 4 y of life, reflecting the natural development of immunity to uncomplicated clinical disease. However, the documented rate of immunity acquisition was slower than expected given the moderate-to-high transmission rate that had been estimated in the area. This may be partly explained by the fact that the study population had prompt access to diagnosis and treatment, which may delay the development of immunity [18,19]. Nonetheless, immunity development against severe malaria and severe anaemia in the group of control participants was faster, and was consistent with the long-standing view that a relatively small number of infections are sufficient to develop reasonably solid protection against the more severe consequences of P. falciparum infection [20].

Malaria chemoprophylaxis with Deltaprim in children aged between 2 and 12 mo caused a significant decrease in the risk of febrile malaria, anaemia, hospital admission, severe malaria, and all causes of hospital attendance during the first year of life. However, the reduction in these endpoints decreased with increasing age, approaching zero towards the end of the first year of life. The cause of this trend is unknown. Compliance did not decrease, and it is unlikely that drug resistance would develop in such a short timeframe. It is possible that the use of a standard recommended dose during the prophylaxis period, which was unlinked to a dose per weight of the child, resulted in sub-therapeutic drug concentrations in older children. A hypothetical reduction with age in the synergy between declining maternally transferred immunity and a moderately efficacious drug may also contribute to the loss of drug efficacy during the first months of infancy. However, the loss in efficacy was also apparent in the latter months of infancy when transplacentally transferred antibodies are not expected to remain [21].

During the entire follow-up period, the difference in deaths was not statistically significant (19 versus 13 deaths, *p* = 0.512). There was an increased number of deaths in the chemosuppressed group (ten deaths versus three, *p* = 0.088) in the 2–4 y age group. However, we were unable to obtain information on the causes and circumstances of those deaths. Given that the number of deaths was low, this difference is most likely to be due to chance, although we cannot rule out a potential and transient increased risk in mortality.

These findings have a number of important implications. Firstly, they lend support to the idea that there are a minimum number of episodes that an individual must experience before effective immunity is developed against mild uncomplicated malaria [22,23]. Secondly, it appears that children who start building immunity after the age of 1 y do so at a slower rate than those who start in infancy, explaining the long duration of the rebound. This difference may, in part, be a reflection of the decreasing EIR in the area. However, it is unlikely to be the main factor involved, as it would imply a rapid change in the transmission intensity that would have been reflected by a rapid drop in the malaria incidence in children receiving placebo—and this did not occur.

Thirdly, the results could be interpreted as suggesting that the benefits of chemoprophylaxis in infancy may be negated by the overall slight increase in the number of malaria episodes in the early years of life. The end of chemoprophylaxis was followed by an immediate increase in the incidence of febrile malaria, well beyond the incidence in placebo recipients—a so-called rebound effect. This increased risk continued for at least 18 mo and was large enough that, by the age of 2 y, the cohorts had experienced a similar number of febrile malaria episodes. Some authors have speculated that shifting the age pattern of disease to the right, in other words delaying the acquisition of immunity to older ages, may pose further hazards by increasing the more severe consequences of P. falciparum [24,25]. Our data do not support this conclusion, but instead suggest that a delayed acquisition of immunity may lead to a small, but not significant, increase in the cumulative number of malaria episodes and, importantly, to a lower CR of severe malaria. A similar pattern was observed for severe anaemia, which is the other major life-threatening complication of malaria in young children. In summary, shifting the age pattern of disease to older age groups does not markedly affect the overall number of mild uncomplicated febrile episodes or lead to an increase in severe malaria, and it is associated with an overall decreased CR of severe anaemia.

Finally, the data provide insight into the effects of age on the clinical manifestations of infection. The observed reduction in the CR of severe anaemia in the intervention group provides a strong indication that malarial anaemia is dependent on the age at which infection takes place: children aged less than 1 y had a higher rate of anaemia than older children, as described by previous research [5]. Furthermore, it appears that this rate is not solely a function of acquired immunity or level of endemicity, but must be related to an age-dependent pathophysiological mechanism. Similarly, the CR of severe malaria was slightly lower in those children who had been previously chemosuppressed. Our data indicate that the risk of progressing from a mild to a severe episode of malaria or anaemia is greater in infants than in older children—a finding that has not been documented previously. Thus, delaying immunity acquisition does not appear to translate into increases in disease severity.

These results show that reducing exposure early in life in a very major way, such as through continuous prophylaxis, can slow down the development of clinical immunity and can lead to a rebound in the risk of disease. However, shifting the age pattern of disease may lead to an overall slightly increased cumulative rate of uncomplicated malaria which was not associated with an increase in severe malaria or severe anaemia. The rebound induced by continuous prophylaxis at this early age has not been documented in other exposure-reduction interventions such as insecticide-treated bednets or when administering two or three treatment doses of anti-malarials at routine immunization contacts during the first year of life (intermittent preventive treatment of malaria in infants) [26]. Finally, the results point at the intrinsic effect of young age in determining the risk of severe anaemia and of severe malaria and the need to target interventions that prevent malaria in young children and, particularly, in infants.

## Supporting Information

Protocol S1 Details of the Statistical Models Used in the Study (85 KB DOC)

## Abbreviations

CI: confidence interval
CR: cumulative rate
EIR: entomological inoculation rate
IDIBAPS: Institut di investigacions Biomediques August Pi i Sunyer
PCV: packed cell volume
PYAR: person-year(s) at risk
SFDDH: St. Francis Designated District Hospital

## References

[pmed-0040242-b001] Ross R (1910). The prevention of malaria.

[pmed-0040242-b002] Stevenson MM, Riley EM (2004). Innate immunity to malaria. Nat Rev Immunol.

[pmed-0040242-b003] Baird JK (1995). Host age as a determinant of naturally acquired immunity to Plasmodium falciparum. Parasitol Today.

[pmed-0040242-b004] Snow RW, Omumbo JA, Lowe B, Molyneux CS, Obiero JO (1997). Relation between severe malaria morbidity in children and level of Plasmodium falciparum transmission in Africa. Lancet.

[pmed-0040242-b005] Schellenberg D, Menendez C, Kahigwa E, Font F, Galindo C (1999). African children with malaria in an area of intense Plasmodium falciparum transmission: Features on admission to the hospital and risk factors for death. Am J Trop Med Hyg.

[pmed-0040242-b006] Greenwood BM, Greenwood AM, Bradley AK, Snow RW, Byass P (1988). Comparison of two strategies for control of malaria within a primary health care programme in the Gambia. Lancet.

[pmed-0040242-b007] Greenwood BM, David PH, Otoo-Forbes LN, Allen SJ, Alonso PL (1995). Mortality and morbidity from malaria after stopping malaria chemoprophylaxis. Trans R Soc Trop Med Hyg.

[pmed-0040242-b008] Otoo LN, Riley EM, Menon A, Byass P, Greenwood BM (1989). Cellular immune responses to Plasmodium falciparum antigens in children receiving long term anti-malarial chemoprophylaxis. Trans R Soc Trop Med Hyg.

[pmed-0040242-b009] Otoo LN, Snow RW, Menon A, Byass P, Greenwood BM (1988). Immunity to malaria in young Gambian children after a two-year period of chemoprophylaxis. Trans R Soc Trop Med Hyg.

[pmed-0040242-b010] Menendez C, Kahigwa E, Hirt R, Vounatsou P, Aponte JJ (1997). Randomised placebo-controlled trial of iron supplementation and malaria chemoprophylaxis for prevention of severe anaemia and malaria in Tanzanian infants. Lancet.

[pmed-0040242-b011] Smith T, Charlwood JD, Kihonda J, Mwankusye S, Billingsley P (1993). Absence of seasonal variation in malaria parasitaemia in an area of intense seasonal transmission. Acta Trop.

[pmed-0040242-b012] Schellenberg D, Menendez C, Aponte J, Guinovart C, Mshinda H (2004). The changing epidemiology of malaria in Ifakara town, southern Tanzania. Trop Med Int Health.

[pmed-0040242-b013] Drakeley C, Schellenberg D, Kihonda J, Sousa CA, Arez AP (2003). An estimation of the entomological inoculation rate for Ifakara: A semi-urban area in a region of intense malaria transmission in Tanzania. Trop Med Int Health.

[pmed-0040242-b014] Hatz C, Abdulla S, Mull R, Schellenberg D, Gathmann I, Kibatala P (1998). Efficacy and safety of CGP 56697 (artemether and benflumetol) compared with chloroquine to treat acute *falciparum* malaria in Tanzanian children aged 1–5 years. Trop Med Int Health.

[pmed-0040242-b015] Alonso PL, Smith T, Schellenberg JR, Masanja H, Mwankusye S (1994). Randomised trial of efficacy of SPf66 vaccine against Plasmodium falciparum malaria in children in southern Tanzania. Lancet.

[pmed-0040242-b016] Spiegelhalter DJ, Thomas A, Best N, Lunn D (2003). WinBugs 1.4 [computer program].

[pmed-0040242-b017] Gelman A (2004). Bugs.R [computer program].

[pmed-0040242-b018] Gatton ML, Cheng Q (2004). Modeling the development of acquired clinical immunity to Plasmodium falciparum malaria. Infect Immun.

[pmed-0040242-b019] Scheller LF, Azad AF (1995). Maintenance of protective immunity against malaria by persistent hepatic parasites derived from irradiated sporozoites. Proc Natl Acad Sci U S A.

[pmed-0040242-b020] Gupta S, Snow RW, Donnelly CA, Marsh K, Newbold C (1999). Immunity to non-cerebral severe malaria is acquired after one or two infections. Nat Med.

[pmed-0040242-b021] Achidi EA, Perlmann H, Salimonu LS, Perlmann P, Walker O (1995). A longitudinal study of seroreactivities to Plasmodium falciparum antigens in Nigerian infants during their first year of life. Acta Trop.

[pmed-0040242-b022] Ntoumi F, Contamin H, Rogier C, Bonnefoy S, Trape JF (1995). Age-dependent carriage of multiple Plasmodium falciparum merozoite surface antigen-2 alleles in asymptomatic malaria infections. Am J Trop Med Hyg.

[pmed-0040242-b023] Trape JF, Rogier C (1996). Combating malaria morbidity and mortality by reducing transmission. Parasitol Today.

[pmed-0040242-b024] Snow RW, Marsh K (2002). The consequences of reducing transmission of Plasmodium falciparum in Africa. Adv Parasitol.

[pmed-0040242-b025] Lengeler C, Schellenberg JA, D'Alessandro U (1995). Will reducing Plasmodium falciparum malaria transmission alter malaria mortality among African children?. Parasitol Today.

[pmed-0040242-b026] Schellenberg D, Menendez C, Aponte JJ, Kahigwa E, Tanner M (2005). Intermittent preventive antimalarial treatment for Tanzanian infants: Follow-up to age 2 years of a randomised, placebo-controlled trial. Lancet.

