# Correction: Age Interactions in the Development of Naturally Acquired Immunity to Plasmodium falciparum and Its Clinical Presentation

**DOI:** 10.1371/journal.pmed.0040359

**Published:** 2007-12-27

**Authors:** John J Aponte, Clara Menendez, David Schellenberg, Elizeus Kahigwa, Hassan Mshinda, Penelope Vountasou, Marcel Tanner, Pedro L Alonso

Correction for:

Aponte JJ, Menendez C, Schellenberg D, Kahigwa E, Mshinda H, et al. (2007) Age Interactions in the Development of Naturally Acquired Immunity to Plasmodium falciparum and Its Clinical Presentation. PLoS Med 4(7): e242. doi:10.1371/journal.pmed.0040242

There was an error in Figure 4A published with this paper. The information contained in the figure is accurate but corresponds to an analysis comparing the cumulative rate of clinical malaria between the groups that received iron supplementation or placebo in this trial. The correct graph of the comparison between the antimalarial chemoprophylaxis and the placebo group is shown here.

**Figure 4:**
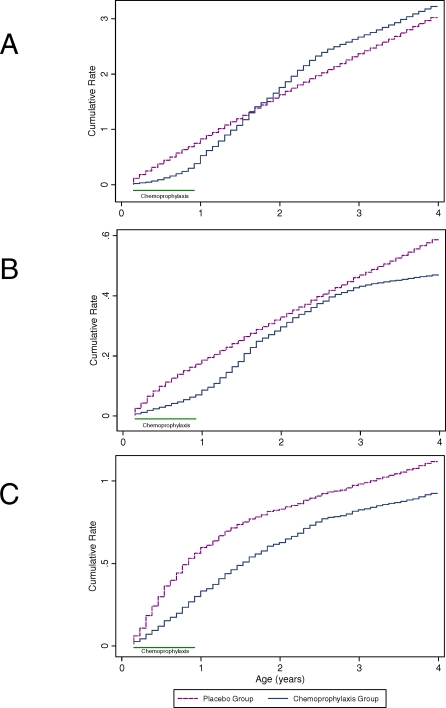
CRs of Clinical Manifestations of Malaria by Group and Age (A) Clinical malaria, (B) severe malaria, and (C) severe anaemia.

