# Supplementary material for: Age Interactions in the Development of Naturally Acquired Immunity to Plasmodium falciparum and Its Clinical Presentation
Source: PLoS Med. 2007 Jul 31;4(7):e242. doi: 10.1371/journal.pmed.0040242 (PMC1950208; doi:10.1371/journal.pmed.0040242)
Supplement: Protocol S1 — (85 KB DOC) [file pmed.0040242.sd001.doc]

## Poisson model of small intervals of time with bayesian autoregressive constrains

Let $n_{i}$ be number of episodes in each age and chemoprophylaxis group combination.We assume it follows a Poisson distribution with mean $\mu_{i}$ . We define the linear part of the model as:

$$\log(\mu_{i})=\log(ptr_{i})+\alpha+\varepsilon_{age_{i}}+\beta_{age_{i}}Chemoprophylaxis_{i}$$

where the offset $ptr_{i}$ is the person time at risk, $\alpha$ is a constant term. $\varepsilon_{age}$is a random effect for age, $\beta_{age}$ is the effect for the chemoprophylaxis at that age.

The $age$ is the variable that codifies the age as months of 28 days ranged from 1 for age 2 months up to 51 for the children with the longest follow-up. The $Chemoprophylaxis$ is a dummy variable with value 0 for the placebo group and 1 for the group that received the chemoprohpylaxis with DeltaprimTM.

The prior $\alpha$ for follows a normal distribution with mean 0 and precision 0.01

The$\beta_{age}$ term is modeled as a mean value $\beta_{0}$ plus a random effect ­ $\nu_{age}$as:

$$\beta_{age}=\beta_{0}+\nu_{age}$$

The prior follows a normal distribution with mean 0 and precision $\tau_{\nu}$ . The autocorrelation is introduced in the definition of the mean values for the priors of to $\nu_{51}$which are defined as

$\rho_{\nu}\nu_{age-1}$ with a precision of $\tau_{\nu}$. The $\rho_{\nu}$ is a term that models the correlation with the previous value of . The $\rho_{\nu}$ has as prior an uniform distribution ranged from -1 to 1 .

Following the same approach, the$\varepsilon$ term is also modeled as a random effect with an autocorrelation prior

$$\varepsilon_{age}=\varepsilon_{0}+\phi_{age}$$

The $\phi_{1}$ prior follows a normal distribution with mean 0 and precision $\tau_{\phi}$ . The priors of $\phi_{2}$ to $\phi_{51}$ are defined as $\rho_{\phi}\phi_{age-1}$ with a precision $\tau_{\phi}$ . The $\rho_{\phi}$ has as prior an uniform distribution ranged from -1 to 1.

The $\tau_{\nu}$ and $\tau_{\phi}$ have as prior a gamma distribution with parameters 0.1 and 0.1.

$\tau_{\nu}$ is defined as $\tau_{\upsilon}^{2}/\left(1-\rho_{\upsilon}^{2}\right)$ and is $\tau_{\phi}$defined as $\tau_{\phi}^{2}/\left(1-\rho_{\phi}^{2}\right)$

The main interest of this analysis is on the relative risk for a given $age$ which is defined as :

$$RR=e^{\beta_{age}}$$

We present the mean value and 95% Credible Intervals (CI) from the posterior distribution of 20000 iterations after a burn in of 15000 iterations.

## Cumulative Rate

The cumulative rate at month is calculated as the sum of the average incidence until month $t$ using the following formula:

$$CR_{t}=\sum_{j=1}^{t} \frac{d_{j}}{ptr_{j}}\Delta t_{j}$$

where $d_{j}$ is the estimated number of episodes in $age_{j}$ from the model described above, $ptr_{j}$is the person time at risk in days for the $age_{j}$ and $\Delta t$ is the time interval for $age_{j}$ which is 28 days.
